# The Efficacy of Combined Alarm Therapy Versus Alarm Monotherapy in the Treatment of Monosymptomatic Nocturnal Enuresis: A Review of Current Literature

**DOI:** 10.5152/eurasianjmed.2022.22311

**Published:** 2022-12-01

**Authors:** Tugay Aksakallı, Ahmet Emre Cinislioğlu, Yılmaz Aksoy

**Affiliations:** 1 Erzurum Regional Training and Research Hospital, Department of Urology, Health Sciences of University, Erzurum, Turkey; 2 Department of Urology, Atatürk University Research Hospital, Erzurum, Turkey

**Keywords:** Enuresis, therapy, children, review

## Abstract

Primary monosymptomatic nocturnal enuresis is a common clinical condition in childhood and affects the psychosocial development of the child. The management of this clinical condition, which includes the preschool and adolescence period, is very important for child development. Diagnostic evaluation should be performed in terms of diabetes mellitus, diabetes inspidus, neurogenic bladder, spinal anomalies, and congenital urogenital system anomalies. Treatment modalities in primary monosymptomatic nocturnal enuresis include enuretic alarm therapy, behavioral therapy, and pharmacological treatments such as desmopressin, tricyclic antidepressants, and anticholinergics. There are also experimental treatments such as percutaneous nerve stimulation, acupuncture, and manual therapy. In this study, we examined randomized controlled studies in the literature that included alarm monotherapy and combined therapy. We aimed to present the efficacy, advantages, and disadvantages of combined treatment with the results of the studies.

**Main Points**

- Enuresis nocturna is a pathology that concerns the general childhood and is associated with the child’s psychosocial development.
- Treatment modalities include behavioral treatments, pharmacological treatments, and combinations thereof.
- Although combination therapies are the methods used by many physicians in clinical practice, the number of studies evaluating the efficacy of combination therapy is very limited in the literature.
- Our review of studies comparing alarm therapy and combined therapy in the treatment of monosymptomatic enuresis nocturna showed that combined therapy may be an option in families who want an early response, but it does not increase the response rate to treatment.

## Introduction

Enuresis nocturna is defined as the involuntary leakage of urine during sleep in children older than 5 years of age without a neurological disorder. Monosymptomatic nocturnal enuresis (MSE) is used to describe enuresis in children without any lower urinary tract symptoms and without a history of bladder dysfunction.^1^

Overall, about 15% of 5-year-olds in the West wet the bed at night to varying degrees, with an annual spontaneous resolution rate of 15% on average. Only 1% to 2% of 15-year-olds still wet the bed.^1^ The possibility of spontaneous recovery in enuresis lasting until this age is very high.^2^

Enuresis is generally thought to result from a maturational delay in the development of bladder control.^3^ In recent years, it has been stated that there is a circadian clock in the kidneys, brain, and bladder, and there are studies on the chronobiology of urination in enuresis.^4^

The main purpose of the evaluation is to detect underlying bowel bladder dysfunction (BBD), posterior urethral valve, spinal dysraphism, and exclusion of diseases such as diabetes mellitus and confirmation that enuresis is indeed monosymptomatic.^5^ If the patient has BBD, this condition needs to be treated before MSE treatment.^6^

### Treatment Modalities

General advice can always be given to children who wet the bed; active treatment should not be started before age of 6 years.^7^ Monosymptomatic nocturnal enuresis is a disease that can resolve spontaneously at an average of 15% each year, observing and following the natural course of the disease may be one of the treatment options.^8^ It has been shown that the mother–child relationship and parental attitude during wetting periods are important in the management of Primary monosymptomatic nocturnal enuresis (PMNE) and a multidisciplinary approach is required.^9^

### Behavioral Therapy

Although well-designed randomized studies of behavioral therapy are lacking, clinical experience suggests that this approach is useful.^10^ Behavioral treatment includes suggestions such as providing motivation for the child, explaining how to urinate without urgency, and controlling fluid intake in the evening. In addition, family education and motivation, which are recommended and emphasized in many childhood psychiatric diseases, are essential.^11,12^ It has been shown that the success of treatment can increase positively with the establishment and long-term motivation of the patient.^13^

### Alarm Therapy

Alarm therapy seems to be a treatment option with the most effective long-term results in the treatment of MSE.^14^ An enuresis alarm is a small device placed under underwear or a bed sheet that contains a wet-sensitive sensor and a stimulus that generates a bell or vibration to wake it up. The family should teach the child to charge the alarm. When the alarm goes off, the child should get up and empty the remaining urine into the toilet, change their clothes and go to bed by setting the alarm again. Since some children cannot wake up after the alarm goes off, their parents should follow up and wake them up at the start of treatment. Interestingly, with the enuresis alarm, patients stay dry without having to get up to pee and having a real sleep through the night. This response is more gradual and sustained than desmopressin. About two-thirds of children remain dry during active treatment and almost half remain dry after treatment is complete.^15^

Success in treated children often begins in the first month, and treatment needs to be continued for 3-6 months for persistent dryness. Treatment should be terminated after the child has been dry for several months.^16^

### Pharmacological Treatment

**Desmopressin. **Desmopressin (1-deamino-8-d-arginine vasopressin) is a synthetic analog of the anti-diuretic hormone released from the posterior pituitary, which decreases urine production by increasing water reabsorption from collector tubes.^17^ The patient group in which desmopressin is most effective is children with nocturnal polyuria, a condition in which nocturnal urine production is greater than 130% of the age-appropriate expected bladder capacity, as defined by the International Children’s Continence Society. Although it has been used in heterogeneous patient populations, the average rate of complete dryness of desmopressin has been reported to be 30%, and a significant reduction rate of nocturnal bedwetting attacks of 40%.^18^

### Anticholinergics

Anticholinergics such as oxybutynin or tolterodine have been shown to be ineffective in the primary treatment of MSE.^19^ The role of combined therapy with anticholinergics is evident in the treatment of children refractory to desmopressin therapy. Oral administration of oxybutynin 5 mg tablet or syrup or 2 mg tolterodine tablet 1 hour before bedtime in enuretic children with decreased bladder capacity at night and/or deterator overactivity is recommended to be given.^20^

Patients using anticholinergic drugs should be followed closely for postvoid residual urine and constipation. Other complications of anticholinergics are dry mouth, blurred vision, flushing, heat intolerance, and mood changes.^21^

### Tricyclic Antidepressants

Tricyclic antidepressants (TCA) have been shown to reduce the REM (Rapid eye movement) period of sleep, stimulate ADH (Antidiuretic hormon) secretion, and relax the detrusor muscle through their weak anticholinergic properties.^22^ The antienuretic properties of these drugs have been shown to affect the kidney and bladder. It is theoretical because its effects are small. A more likely effect is noradrenergic stimulation to the brainstem, specifically to the locus coeruleus.^23^

Although TCA treatment is more successful in enuretics resistant to other treatments, older children, and those with low bladder capacity, treatment success is lower in those with constipation and daytime incontinence. Although side effects are rare TCAs, it has been shown that urinary retention may develop in some antidepressants.^24^ About 5% of treated children experience neurological symptoms such as irritability, personality changes, and sleep disturbances.^25^ Experimental treatments such as acupuncture, pelvic floor muscle training, and acupressure neuromodulation have also been reported in various studies.^26-28^

In our study, we reviewed the literature studies comparing the alarm treatment applied as monotherapy and the combined treatment in which alarm and pharmacological treatment were applied together. With the results of these studies, we aimed to determine the efficacy, advantages, and disadvantages of combined therapy.

## Clinical and Research Consequences

Our study includes a review of the comparative results of treatments applied in patients with MSE in the literature. Before 2022, related studies were found by searching PubMed, ResearchGate, and Medline databases for the keywords “monosymptomatic nocturnal enuresis treatment,” “children,” “comparative study,” “alarm therapy.” In this way, 9 related studies were identified. Of the studies, 2 were excluded because they were reviews and 1 was about behavioral therapy.

**Figure 1.**
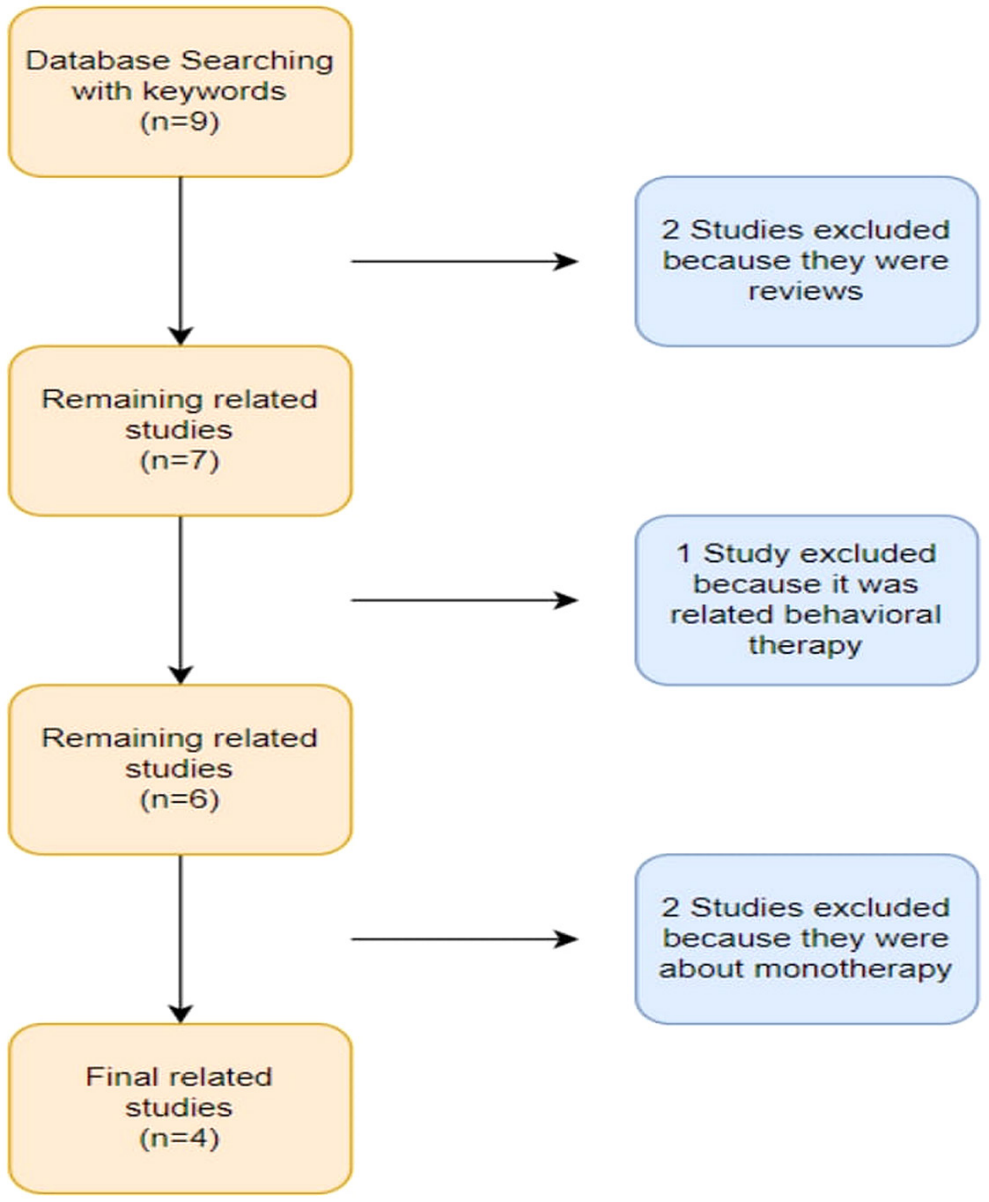
Study design.

When the full text of the articles was read, 2 studies were excluded because they were a comparison of monotherapies. Four studies involving a total of 259 patients were included in the review (Figure 1).

### Methodological Quality of Articles

Included articles were evaluated in The Joanna Briggs İnstitude (JBI) Critical Appraisal Checklist for randomized controlled trials, which consists of 13 questions. All studies methodologically met the inclusion criteria.

In 4 studies, patients were aged 5-15 years, and patient numbers ranged from 19 to 105. The details of the studies are presented in Table 1. In studies, the efficacy of desmopressin, enuretic alarm, and imipramine treatments was investigated in a prospective randomized manner. Vogt et al^29^ shared the clinical study including the results of the combined treatment after primary treatment failure, Tuygun et al^30^ shared the study examining the enuretic alarm treatment applied after unsuccessful desmopressin treatment, and the other 2 studies shared the results of monotherapy and combined treatment in patients randomized into 3 groups.

### Findings from the Comparison of Combined Alarm Therapy and Alarm Monotherapy

In the study of Vogt et al^29^ in which they started with desmopressin and alarm monotherapy in 43 patients, they applied desmopressin and alarm combined treatment to 30 patients who did not respond to treatment at the third month in their evaluation and showed that dryness was achieved with 73%.^29^ The most conspicuous limitation of this study is the low sample size.

In the study they planned as 3 groups, Tuygun et al^30^ started enuretic alarm treatment in 35 patients in group 1 and desmopressin treatment in 49 patients in group 2 and compared the success rates. In the sixth month evaluation, the success rates for group 1 and group 2 were 54.28% and 26.53%, respectively (*P* = .007). Enuretic alarm therapy was started in 19 patients in group 2, for whom the treatment was unsuccessful. At the end of 6 months, no statistical difference was found in the comparison of group 1 and group 3 (*P* = 1.000).^30^ In this study, it was determined that previous pharmacotherapy did not change the efficacy of enuretic alarm.

Naitoh et al^31^ compared the results of 3 groups of 105 patients who received alarm monotherapy with desmopressin and imipramine in addition to alarm therapy. While the improvement rate, defined as a reduction in wet nights of more than 50%, did not change at 3 months, it was found to be higher and statistically significant in the combined treatment of desmopressin (*P* = .059) and imipramine (*P* = .082) at 6 months. The cure rate that was defined as 0-1 wet nights/month was found to be 30%, 20%, and 21% higher in the monotherapy group. However, they reported that response time to treatment was shorter in combined treatments.^31^ As a result, the authors stated that combined therapy may be an appropriate option in the patient group who did not respond to alarm treatment in 3 months and in families who want to achieve an early response.

Fai-Ngo Ng et al^32^ compared 3 groups of 105 patients who were started on alarm monotherapy, desmopressin, and alarm plus desmopressin treatments. The rate of 0-1 wet/week in the last 4 weeks of combined treatment, defined as complete responders, was found to be the highest with 62.5%. A greater than 50% reduction in the number of wet nights, defined as the partial response, was found to be the highest in the desmopressin group. There were no significant differences in the early relapse rate (*P* = .31) and total relapse rate (*P* = .109) between the complete responders and the partial responders, but interestingly, all complete responders in the alarm group remained dry after the treatment was stopped. The study showed that sustained complete plus partial response rates at 3 months after a short course of treatment ranged from 40.6% for combined therapy to 28.6% for alarms and 21% for desmopressin alone.^32^ As a result, they reported that combined treatment can be started for families who are willing to achieve a faster response.

### Limitations of Review

This review has some limitations arising from the studies in the literature.

- In studies obtained as a result of screening, the sample contains a small number of patients.
- The factors determined in response to treatment have different elements in each study.
- In only 2 of the studies, enuretic alarm monotherapy and combined treatment groups were determined during randomization. In the other 2 studies, combined treatment was started after treatment failure.

## Conclusion

Despite limitations, our study found that combined therapy did not show superiority over alarm monotherapy in response to treatment. However, it is understood that combined therapy should be considered as an option in the initial treatment in families who want to achieve an early response. The fact that the combined treatment provided the highest success rate in the study of Fai-Ngo Ng et al^32^ shows the need for randomized controlled studies with larger samples on this subject.

## Figures and Tables

**Table 1.** Characteristics of Studies

Authors, Year, and Country	Study Design and Sample Size	Length of Study	Success Criterion	Results of Study
Vogt et al (2010) Germany	RCT Age: 5-15 years (n = 30)	6 months (3-month monotherapy, 3-month combined therapy)	0-1 wet nights/month	No statistically significant difference between alarm and desmopressin. After combined therapy, success rate came up to 73%.
Tuygun et al (2007) Turkiye	RCT Age:6-13 years (n = 19)	6 months	>90% dry nights/month	No statistically significant difference between alarm and desmopressin. Combined therapy did not improve the success rate.
Yasuyiki et al (2005) Japan	RCT Age: 6-13 years (n = 105)	6 months	İmprovement: Reduction >50% wet nights Cure: 0-1 wet nights/month	Improvement rate in combined groups greater than monotherapy. However, alarm monotherapy cure rate is greater than combined therapy.
Fai-Ngo ng et al (2005) Hong Kong	RCT Age: 7-15 years (n = 105)	6 months (3-month treatment, 3 follow-up)	Complete response: 0-1 wet/week in the last 4 weeks of treatment. Partial responders: greater than 50% reduction in the number of wet nights.	Combined therapy showed immediate short-term response, while alarm therapy gave more gradual and sustained improvement.

RCT, randomized controlled trials.
